# UV LED lighting for automated crystal centring

**DOI:** 10.1107/S0909049510028670

**Published:** 2010-11-05

**Authors:** Leonard M. G. Chavas, Yusuke Yamada, Masahiko Hiraki, Noriyuki Igarashi, Naohiro Matsugaki, Soichi Wakatsuki

**Affiliations:** a Structural Biology Research Center, Photon Factory, High Energy Research Organization (KEK), 1-1 Oho, Tsukuba, Ibaraki 305-0801, Japan

**Keywords:** UV light, LED, macromolecular crystallography, automation, high throughput, crystal centring

## Abstract

A direct outcome of the exponential growth of macromolecular crystallography is the continuously increasing demand for synchrotron beam time, both from academic and industrial users. As more and more projects entail screening a profusion of sample crystals, fully automated procedures at every level of the experiments are being implemented at all synchrotron facilities. One of the major obstacles to achieving such automation lies in the sample recognition and centring in the X-ray beam. The capacity of UV light to specifically react with aromatic residues present in proteins or with DNA base pairs is at the basis of UV-assisted crystal centring. Although very efficient, a well known side effect of illuminating biological samples with strong UV sources is the damage induced on the irradiated samples. In the present study the effectiveness of a softer UV light for crystal centring by taking advantage of low-power light-emitting diode (LED) sources has been investigated. The use of UV LEDs represents a low-cost solution for crystal centring with high specificity.

## Abstract 2

A low-cost light-emitting diode (LED) UV source has been developed for facilitating macromolecular sample centring in the X-ray beam.

## 1. Introduction

The last decade has been marked by an upturn in macromolecular crystallography. Structural genomic projects around the globe have greatly assisted the expansion of highly efficient and exportable methods for sample preparation and crystallization that have led to the structure determination of a large quantity of molecules (Joachimiak, 2009 ▶). The exponential increase in the number of coordinates deposited in the Protein Data Bank reflects well the recent advances in macromolecular structure phasing and refinement methods, and is paralleled by an equally growing number of synchrotron beam-time requests. The science that was once dedicated to experts is now approachable by the larger community, and even a novice can solve a structure in no time. Notably, owing to third-generation synchrotrons coupled with robotics and decision-making software, classical data acquisition is now­adays recorded in minutes, allowing the high-throughput screening of a maximum number of samples in a minimum amount of time. New approaches such as structural-based drug design are now emerging and largely used by pharmaceutical companies, mostly implemented within their workflow towards drug discovery (Tickle *et al.*, 2004 ▶).

**Figure 1:**
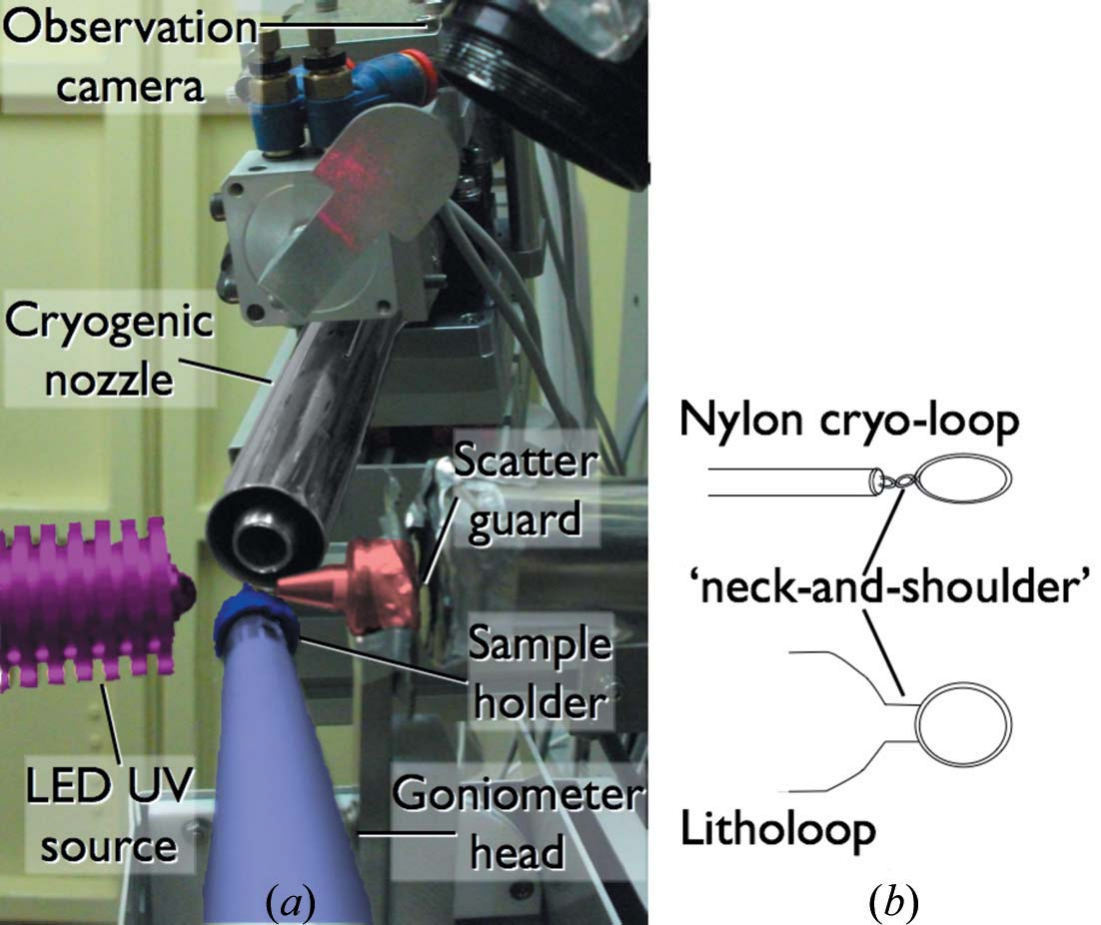
(*a*) Experimental set-up at the PF protein crystallography beamlines. The beam stopper was removed for a better understanding. (*b*) Schematic representation of sample holders commonly used at PF.

Since synchrotron radiation diffraction experiments on protein crystals are becoming highly robotized routines, to understand all the steps of the automation procedure would greatly assist setting-up better experimental protocols, necessary for even faster data acquisition and shorter beamline access time. Most of the macromolecular crystallography beamline diffractometers are used in a ‘classical’ set-up, consisting of a goniometer with (*x*, *y*, *z*)-axis motors to stably orient the sample at the beam position; a cryogenic stream nozzle to keep the samples at low temperatures; a scatter-guard near the sample to provide a small aperture and to minimize scattering from the incoming beam; and a beam stopper to block the direct beam that would damage the X-ray detector (Fig. 1 ▶). When using automated procedures, the first step of mounting the sample on the goniometer head is generally assumed by a multi-axis robot that will pick up the sample from its storage location and transfer it to the goniometer, with attention given to preserving the low temperature of the crystal. At the Photon Factory (PF), this operation is conducted by the PF automated mounting (PAM) system equipped with Gemini double tongues that allow an optimized sample exchange within 10 s (Hiraki *et al.*, 2008 ▶).

To implement a fully automated procedure for high-throughput experiments, the sample is required to be correctly positioned in the X-ray beam. Most of the diffraction experiments use sample holders of pre-defined shapes that can be targeted for aligning the sample using approaches such as the cryoloop centring method (Karain *et al.*, 2002 ▶). Although fast and easily implemented, this method suffers from its simplicity as it is not highly accurate with regard to the crystal position. As a direct consequence, small crystalline forms and multi-crystals within a single sample holder cannot be clearly differentiated. To tackle this issue, independent algorithms have been developed based on various aspects particular of the loaded samples. Most of the approaches focus on increasing the contrast between the crystal and its surroundings, notably by making use of particular illuminations such as backlight illumination (Muchmore *et al.*, 2000 ▶), infrared (Snell *et al.*, 2005 ▶) or ultraviolet (Forsythe *et al.*, 2006 ▶). A non-exhaustive list of other techniques include X-ray diffraction centring (Song *et al.*, 2007 ▶), X-ray fluorescence from crystals potentially containing anomalous scatters (Karain *et al.*, 2002 ▶), and a feature-scoring system (Lavault *et al.*, 2006 ▶).

In the past few years new advances in light-emitting diode (LED) developments have been marked by the appearance of powerful sources at shorter wavelengths, notably in the spectrum of the UV (McGuinness *et al.*, 2004 ▶). Resulting directly from these new technologies, a growing interest in the capacity of UV light for crystal identification is now emerging (Gill, 2010 ▶; Dierks *et al.*, 2010 ▶). In the present study the potential of UV LED sources for crystal centring was investigated, the objective being to implement low-cost and non-destructive UV lights at all PF protein crystallography beamlines. When properly adjusted, UV illumination provides an efficient recognition of crystalline objects with high reproducibility.

## 2. Instrumentation

The standard set-up at PF protein crystallography beamlines is represented in Fig. 1 ▶. The sample holder is fixed onto the air-bearing goniometer head by a magnet. The cryogenic nozzle is motorized to allow the PAM double tongues to have access to the goniometer head while keeping the temperature at the sample below 110 K. In the present set-up the UV LED light source is oriented in the beam direction, located 10 mm from the sample position and parallel but not co-axial to the observation camera (Fig. 1*a* ▶). To gain space and to take advantage of existing motors, future developments will feature the UV LED source parallel and co-axial to the cryogenic nozzle.

The UV LED sources (U-VIX Co.) were calibrated near 265 nm and 280 nm for low- and high-power LEDs. The measured wavelengths were 268.8 (±4) nm and 283.7(±4) nm for the high-power LEDs, and 284.3 (±4) nm for the low-power LED. Measurements of the power density received at the sample position were performed on a C9536/H9535 UV light detector (Hamamatsu) optimized at 280 nm wavelength. For all the measurements the UV source was located at 10–12 mm from the sample position, and the measurements were taken at 200 µm steps through an aperture of 200 µm.

## 3. Centring procedure

The proposed centring procedure using UV LED light is performed in two major steps: (i) identification of the sample holder followed by its alignment at the beam position, (ii) UV illumination of the sample and precise centring of the highlighted crystal.

### 3.1. Automated sample holder recognition and centring

**Figure 2:**
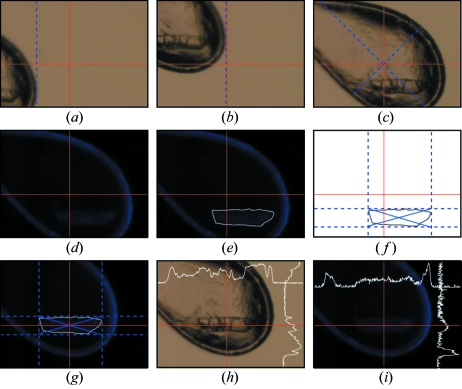
Sequential centring of the sample holder (*a–c*) and the crystal (*d–g*). The loop centring is performed by applying an edge recognition method (*a–b*) followed by mask recognition and centre of mass calculation (*c*). The crystal is centred after UV illumination (*d*), contour recognition (*e–f*) and centring (*g*). The intersection at the red lines represents the beam centre. The blue dotted lines representing the boxes for centre of mass calculation were arbitrarily added for a better understanding of the steps in the centring process. The higher recognition contrast for the UV illuminated crystals is highlighted by comparing the centred crystal under normal light (*h*) and UV light (*i*), together with horizontal and vertical scans along the red lines.

Most of the protein crystallography beamline users at PF make use of commercially available sample holders, such as nylon cryoloops or litholoops (Fig. 1*b* ▶). The recognition procedure of the sample holder is therefore reduced to a pattern match screening of pre-defined shapes, as described elsewhere (Karain *et al.*, 2002 ▶). Briefly, a series of images is collected at fixed angles while the sample holder is rotated around the ω rotation axis. For each image the tip of the sample holder is first detected (Fig. 2*a* ▶) and translated to the beam position (Fig. 2*b* ▶). A pattern recognition cross-correlation of a mask approximating a feature common to both nylon loops and litholoops, the so-called ‘neck and shoulders feature’, is then applied to define the borders of a box that will represent the volume of the sample holder. Its centre of mass is then translated and aligned at the X-ray beam position (Fig. 2*c* ▶).

When non-standard sample holders are used, or when the pattern match is not successful, the same edge detection algorithm is applied to identify the tip of the sample, and the overall shape of the object is assumed to be spherical with a diameter 1.5 times the widest dimension of the observed sample.

### 3.2. UV-based crystal centring

The present procedure aims at a precise crystal centring carried out by collecting images of the centred sample holder illuminated by UV emanating from a LED source. A well known issue with UV light is its property to affect biological samples, notably DNA base pairs, and sometimes proteins (Nanao & Ravelli, 2006 ▶). In order to minimize the possible damage resulting from the irradiation, only very short pulses of UV light are emitted at one time, perfectly synchronized with the image capture. To reduce any background illumination, all the lights in the experimental hutch are automatically switched off when starting the centring. The sample appears highlighted over a black background, sometimes surrounded by the shiny sample holder such as in the case of nylon cryoloops (Fig. 2*d* ▶). Prior to detecting the precise location of the crystal, the contour of the sample holder is removed based on the so-called Model-based Automated Crystal Detection (MaCyD) algorithm (Pothineni *et al.*, 2006 ▶). The advantage of adapting MaCyD to the picture of the UV-exposed sample holder is that with the loop being well visible its boundaries can be effectively removed. Coupled with an edge detection algorithm based on the Laplacian of Gaussian filtering, the contour of the crystal can be clearly identified (Fig. 2*e* ▶). The final step consists of determining the crystal size by delimiting the external edges of the crystal (Fig. 2*f* ▶) followed by calculation of its centre of mass and alignment to the beam position (Fig. 2*g* ▶). The contrast between UV and ambient light illumination can be observed by comparing Figs. 2(*h*) and 2(*i*) ▶. Notably, while the crystal cannot be distinguished from the surrounding background and shadows under normal light illumination on the horizontal and vertical scans (Fig. 2 ▶ *h*), the UV illumination clearly allows the crystalline form to be dissociated from the noise level [scans in Fig. 2(*i*) ▶].

## 4. Results

### 4.1. Exposure time

**Figure 3:**
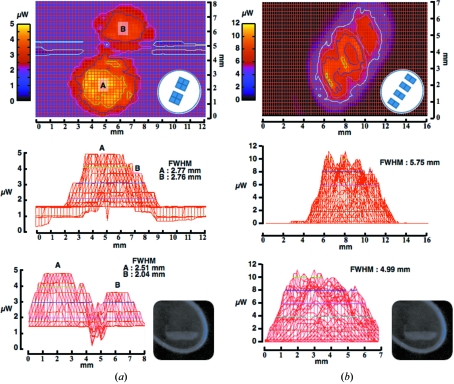
Intensity beam profile of the UV emanating from the high-power LEDs. The top panels represent colour-coded beam intensities over a two-dimensional screening, with the schematic representation of the LED arrangement at the bottom right. The middle and bottom panels are transversal views. (*a*) The measured UV was 268.8 nm, with the two blocks arrangement within the LED resulting in two major peaks. (*b*) The measured UV was 283.7 nm, with an elongated and homogeneous distribution of the beam. UV-illuminated crystals are represented as insets.

The potential of UV light to induce conformational changes within the irradiated crystal is of major concern when using UV as a source for centring. Nanao & Ravelli (2006 ▶) showed that a power density of about 0.1 mW in a 150 µm spot was enough to cause local modifications in protein crystals. For this reason great care was given to the calculation of the minimum dose necessary for crystal centring. In the set-up shown in Fig. 1(*a*) ▶ the dose limit potentially absorbed by the sample is calculated to reach 16 µW in a 200 µm spot for 1 s exposure (Figs. 3 ▶ and 4 ▶). In all of the tested cases a single 300 ms exposure of the sample at three different angles was sufficient to find the precise coordinates for the centre of mass. Using this method, by keeping a short exposure time for a limited number of images, a single crystal is only irradiated by an accumulated dose of about 50 µW, unlikely to induce internal structural modifications (unpublished data).

### 4.2. LED arrangement

**Figure 4:**
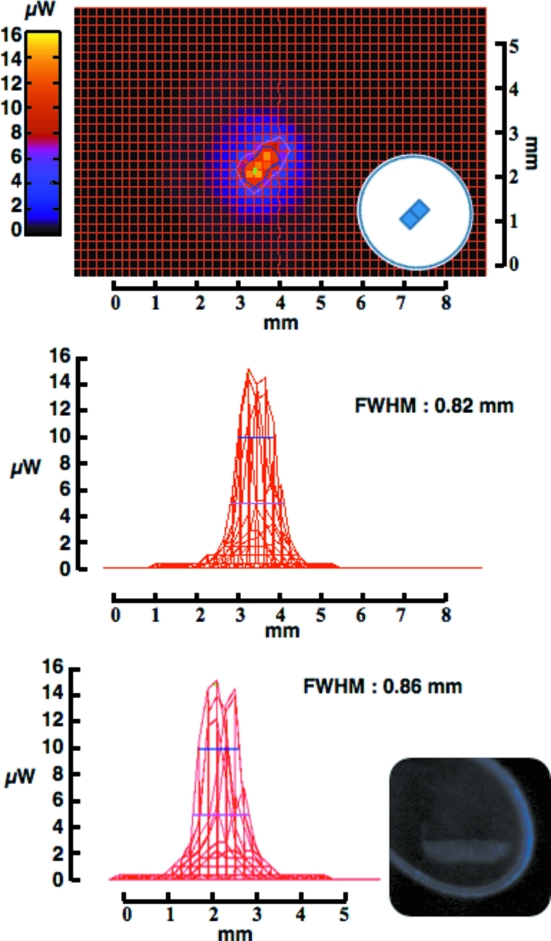
Intensity beam profile of the UV emanating from the low-power LED. Panels are as in Fig. 3 ▶. The measured UV was 284.3 nm, with a sharp and homogeneous beam distribution.

To increase the chances of differentiating the crystal from its surroundings, several LED types were tested for their property in illuminating the sample. Three types of LEDs, different in their arrangements of the internal chips, were screened (Figs. 3 ▶ and 4 ▶). The two high-power LEDs [Fig. 3(*a*) and 3(*b*) ▶] are made of four sets of chips compared with the low-power LED (Fig. 4 ▶). All the LEDs were placed at 10–12 mm from the sample position. As shown in Fig. 3 ▶, the high-power LEDs are not as focused as the low-power LED, resulting in a broader distribution of the emanating UV while the maximum power density remains approximately homogeneous over 5 mm^2^. In addition, the differentiation of the sample from the background is not particularly affected when using either of the LED types (inset in Figs. 3 ▶ and 4 ▶). Taken together, owing to the difficulties in focusing the UV light emanating from the LED sources, it remains challenging to precisely target small objects as well as laser sources would. Nevertheless, the broader spectrum at lower density makes LED sources a proper choice for global illumination of the sample, with no clear difference for visualizing the crystals (inset in Figs. 3 ▶ and 4 ▶).

### 4.3. UV wavelength

Two different wavelengths have been tested, 268.8 nm and 283.7 nm. In both cases UV illumination resulted in the visualization of the sample without any striking difference between the two wavelengths (data not shown). A comparison of the amount of possible damage induced by the exposure at both wavelengths, in order to decide which UV energy would be favourable for crystal centring without affecting the internal chemistry of the sample, is yet to be investigated. This work is now under investigation at our beamlines.

## 5. Conclusions

To achieve automated crystal centring, the two necessary requirements are to obtain a clear image of the crystal within the sample holder and to properly identify the crystal shape. In the present investigation the potential of UV LED sources for macromolecular crystal centring has been described. Crystalline objects are clearly identified, with a higher contrast with the surrounding buffer when compared with ambient light illumination, resulting in a more efficient edge-recognition procedure for characterizing the crystal edges. When properly used, the low power emanating from these LED sources can be applied for crystal centring with non-destructive effects, even though further investigation is required to comprehensibly understand the potential influence of such LEDs on the macromolecular structures. Although still to be finalized, the present algorithm for crystal centring will shortly be incorporated into the PF protein crystallography beamline control software UGUI/S. As new approaches for crystal mounting are coming forth, such as the loop-free mounting procedure (Kitago *et al.*, 2010 ▶), universal centring methods that would target any object of various size and shape need to be implemented. The capacity of soft UV to specifically highlight biological objects makes such a light source a propitious target for future developments.
